# Robot-assisted laparoscopic ileal ureter replacement with extracorporeal ileal segment preparation for long ureteral strictures: a case series

**DOI:** 10.1186/s12893-022-01885-5

**Published:** 2022-12-21

**Authors:** Shubo Fan, GuanPeng Han, Zhihua Li, Xiang Wang, Xinfei Li, Shengwei Xiong, Dan Li, Jun Zhang, Chang Meng, Peng Zhang, Kunlin Yang, Xuesong Li, Liqun Zhou

**Affiliations:** 1 Department of Urology, Peking University First Hospital, Institute of Urology, Peking University, National Urological Cancer Center, No. 8 Xishiku St, Xicheng District, Beijing, 100034 China; 2 Nursing Department, Peking University First Hospital, No. 8 Xishiku St, Xicheng District, Beijing, 100034 China; 3 Department of Urology, Emergency General Hospital, No. 29, Xibahenanli, Chaoyang District, Beijing, 100028 China

**Keywords:** Extracorporeal ileal segment preparation, Long ureteral strictures, Robotic ileal ureter, Surgical technique

## Abstract

**Background:**

Complete intracorporal robotic ileal ureteric replacement is challenging. We aimed to present the surgical technique of robotic ileal ureter replacement with extracorporeal ileal segment preparation for long ureteral strictures.

**Methods:**

From March 2019 to March 2021, 18 patients underwent robotic ileal ureter replacement with extracorporeal ileal segment preparation by one experienced surgeon. The demographic, perioperative, and follow-up data were recorded. Success was defined as the resolution of the presenting symptom, a stable estimated glomerular filtration rate and unobstructive drainage on imaging examination.

**Results:**

All 18 surgeries were successfully completed without conversion. The median length of the intestinal tube used was 20 (12–30) cm. The median operative time was 248 (170–450) min, the median estimated blood loss was 50 (10–200) ml, and the median postoperative hospital stay was 7 (5–27) days. At a median follow-up of 16 (13–28) months, all patients were symptom-free. No or mild hydronephrosis was confirmed in 17 patients; 1 patient had moderate hydronephrosis without peristalsis of the ileal ureter. The renal function was stable in all patients. The overall success rate was 100%. Postoperative complications, including 4 cases of urinary infections (Grade I), 1 case of an incision hernia (Grade I), 4 cases of kidney stone formation (Grade I), 6 cases of metabolic acidosis (Grade I), 4 cases of incomplete ileus (Grade II), and 1 case of an incision infection (Grade IIIb).

**Conclusions:**

Robot-assisted laparoscopic ileal ureter replacement with extracorporeal ileal segment preparation is safe, feasible, and effective for the treatment of long ureteral strictures, especially in high-volume tertiary referral centers with extensive robotic surgery experience capable of managing severe peri-operative complications.

**Supplementary Information:**

The online version contains supplementary material available at 10.1186/s12893-022-01885-5.

## Background

Long ureteral strictures present a reconstructive challenge for the urologist. Several tissue substitution techniques have been reported when a primary ureteroureterostomy was unsuitable, such as oral mucosa grafts, and appendiceal, pelvic, and Boari flaps [1–5]. Auto-transplantation has excellent long-term results, while the technique is accompanied by a high perioperative morbidity rate. Ileal ureter replacement is still considered to be the ultimate solution, especially for extremely long strictures.

Since ileal ureter replacement was performed for the first time in 1906 and was popularized by Goodwin in 1959 [6], ileal ureter replacement has been confirmed to be a reasonable surgical option with good long-term outcomes [7]. With the development of minimally invasive techniques, the first laparoscopic procedure was reported in 2000 [8] and was shown to have significant benefits in postoperative recovery compared with open ileal ureter replacement [9]. Nevertheless, laparoscopic ileal ureter replacement is daunting to most urologists because of the technical complexity of intracorporal cutting and suturing.

The popularization of a robotic platform has been helpful. The first robot-assisted laparoscopic procedure was performed by Wagner in 2009 [10], complete intracorporeal procedures were introduced in 2014 [11, 12], and several case reports have demonstrated the feasibility of the robotic procedure in recent years [13–17]. Complete intracorporal robotic ileal ureteric replacement presents a significant surgical challenge.

Similarly, the completely intracorporeal reconstructive part is challenging and time-consuming for radical cystectomy. Extracorporeal assistance through a small incision is acceptable in laparoscopic and robotic radical cystectomy, which ensures the advantage of minimally invasive surgery and decreases the overall operative time and costs [18, 19].

To simplify this procedure of robotic ileal ureteric replacement, we describe a technique of robotic ileal ureter replacement with extracorporeal ileal segment preparation and share the initial outcomes involving our first 18 cases. Indeed, this study is the largest series report of robot-assisted laparoscopic ileal ureter replacement.

## Methods

Eighteen patients underwent robot-assisted laparoscopic ileal ureter replacement with extracorporeal ileal segment preparation by an experienced surgeon between March 2019 and March 2021. According to our management strategy for ureteral strictures, the ileal ureter was considered when simple anastomosis, renal pelvic flap, appendiceal flap, and lingual mucosa grafts could not satisfy the need for repair [20]. The clinical data, including demographics, surgery details, perioperative records, complications, and patient outcomes, were prospectively collected in our Reconstruction of the Urinary Tract: Technology, Epidemiology and Result (RECUTTER) database. Success was defined as the resolution of the presenting symptom and a stable estimated glomerular filtration rate (eGFR) and unobstructive drainage on imaging examination. All procedures performed in this study were in accordance with the Declaration of Helsinki (as revised in 2013) and approved by the Ethics Committee of Peking University First Hospital (approval number: 2019134).

### Surgical technique

#### Position and trocar distribution

**Fig. 1:**
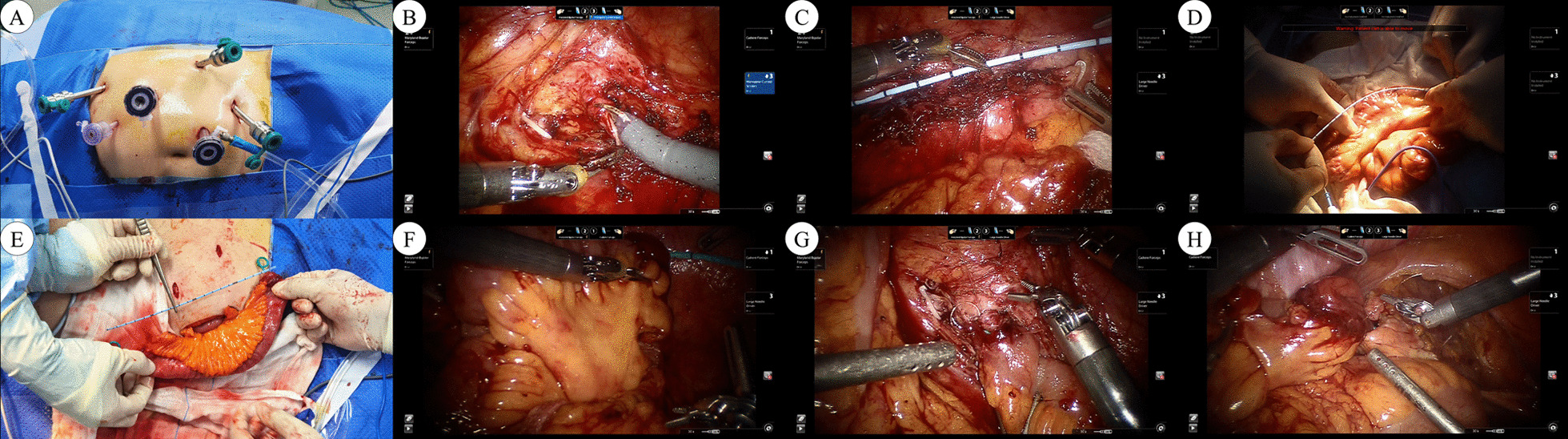
Surgical technique of robot-assisted laparoscopic ileal ureter replacement with extracorporeal ileal segment preparation. **A** Port placement. **B** Ureterectomy. **C** Measurement of the distance from the proximal ureteral transverse section to the bladder dome. **D** The ileal segment (necessary length acquired by the stent measurement) was isolated. **E** The mesentery of harvested bowel segment was incised parallel to the axis of the bowel approximately 3 cm on both sides. **F** For left-sided procedures, the ileal segment was brought into the retroperitoneal cavity through a mesenteric window in the sigmoid colon. **G** The pyeloileal anastomosis. **H** The ileovesical anastomosis

After the induction of general anesthesia, a 20# three-way catheter was inserted. Patients were first placed in a 60° recumbent position for the intracorporeal dissection and ureterectomy. A transperitoneal four-armed technique was used (Fig. 1A) (Additional files 1 and 2).

#### Intracorporeal dissection and ureterectomy

The colon was dissected medially at the line of Toldt and ureterolysis was carried out to mobilize the ureter with dissociation cephalad to the renal pelvis. Care was taken to preserve sufficient periureteral tissue to ensure an adequate blood supply. The ureter was divided proximal to the ureteral lesion, and the ureterectomy was performed (Fig. 1B). Then, a ureteral catheter with a scale was used to measure the distance from the proximal ureteral transverse section to the bladder dome (Fig. 1C). For left-sided procedures, a mesenteric window was needed.

#### Extracorporeal ileal segment preparation

After undocking the robot, the patient was re-positioned to a 40° recumbent position through the horizontal tilt of the operating table for extracorporeal bowel preparation. An approximate 5-cm incision was made around the umbilicus, then the ileal segment (necessary length acquired by the ureter catheter measurement) was isolated approximately 20 cm proximal to the ileocecal valve using monopolar shears (Fig. 1D). The continuity of the bowel was restored with a gastrointestinal stapler in a side-to-side manner. The butt end of the repair was embedded in two layers, and the mesentery was closed. A thorough bowel preparation was performed using diluted povidone iodine to reduce bacteriuria and bacteremia. To straighten the harvested bowel segment, the mesentery was incised parallel to the axis of the bowel segment approximately 3 cm bilaterally, paying attention to retaining the blood supply (Fig. 1E). An anti-reflux nipple was created at the distal end of the isoperistaltic ileal segment and a 7F ureteral stent was inserted and fixed to prevent dislocation, as we have previously described [21].

#### Intracorporeal anastomosis

After the patient was replaced with a 60° recumbent position, we docked the robotic arm and re-established the pneumoperitoneum. For right-sided procedures, the ileal segment was easily placed in the retroperitoneum. For left-sided procedures, the ileal segment was brought into the retroperitoneal cavity through a mesenteric window in the sigmoid colon (Fig. 1F). Adequate spatulation of the proximal ureteric stump or renal pelvis was performed, which allowed for easy adaptation of the ureteric stump to the ileal diameter. Pyeloileal anastomosis was performed in an end-to-end manner with running sutures (Fig. 1G). The bladder was completely mobilized medially off the pelvic sidewall, and the anti-reflux nipple was anastomosed to the bladder at the superolateral wall with running sutures (Fig. 1H). Two drains were placed near the pyeloileal and ileovesical anastomoses. The incision was closed after ensuring good hemostasis.

#### Postoperative management and follow-up

A liquid diet was initially necessary after surgery, then a full diet was gradually introduced. The drainage tube was usually removed 4–6 days after surgery. The three-way catheters were removed 2 weeks after surgery. The nephrostomy tube and the ileal stent were typically removed 2 months postoperatively, then cine magnetic resonance urography (cine MRU) was performed. Follow-up mainly included clinical assessment, serologic testing, renal ultrasonography, and venous blood gas analysis every 3 months postoperatively. Hydronephrosis was graded using ultrasound according to the scale recommended by the Society for Fetal Urology [22]. Complications were categorized according to the Clavien-Dindo classification system.

#### Statistical analysis

Data were analyzed using SPSS 19.0 (IBM Corporation, Armonk, NY, USA). Measurement data are expressed as the median (range). Enumeration data are expressed as numbers (percentage).

## Results

As shown in Table 1, there were 11 males and 7 females, with a median age of 48.5 (25–74) years. The median BMI was 26 (19–32) kg/m^2^. All patients presented with a long ureteral stricture confirmed by preoperative imaging in which 11 were left-sided and 7 were right-sided. Eleven patients presented with flank pain, 2 presented with fever, and 5 were asymptomatic. The median preoperative eGFR was 85 ^(^48–113) ml/min/1.73 m^2^.

**Table 1:** Demographics of the patients

Variable	Value
Patients, *n(%)*	18 (100%)
Age (years), median (range)	48.5 (25–74)
Sex (male/female), *n(%)*	11/7 (61%/39%)
BMI (kg/m^2^), median (range)	26 (19–32)
Laterality, left/right, *n(%)*	11/7 (61%/39%)
Etiology, *n(%)*	
Stenosis after ureteroscopic laser lithotripsy	10 (55.5%)
Ureteral rupture due to ureteroscopic lithotripsy	3 (17%)
Gynecologic surgery and radiation for cervical cancer	1 (5.5%)
Multiple ureteral polyps	1 (5.5%)
Ureteral tuberculosis	1 (5.5%)
Ureteritis for unknown reasons	2 (11%)
Presenting symptoms, n (%)	
Flank pain	11 (61.1%)
Fever	2 (11.1%)
Asymptomatic	5 (27.8%)
Preoperative percutaneous nephrostomy, n (%)	16 (88.9%)
Preoperative double-J stent insertion, n (%)	1 (5.5%)
Preoperative eGFR (mL/min/1.73 m^2^), median (range)	85 (48–113)

As shown in Table 2, all surgeries were successfully performed without laparoscopic or open conversion. The median length of the ileal ureter was 20 (12–30) cm, the median operative time was 248 (170–450) min, and the median estimated blood loss was 50 (10–200) ml. The median time until the patients tolerated liquid and regular diets were 4 (1–7) and 5 (3–13) days, respectively. The median hospital stay was 7 (5–27) days.

**Table 2:** Surgical details and follow-up data

Variable	Value
Length of the ileal ureter (cm), median (range)	20 (12–30)
Operative time (mins), median (range)	248 (170–450)
Estimated blood loss (mL), median (range)	50 (10–200)
Time to liquid (days), median (range)	4 (1–7)
Time to regular diet (days), median (range)	5(3–13)
Postoperative hospital stay (days), median (range)	7 (5–27)
Follow up (months), median (range)	16 (13–28)
No symptoms, *n(%)*	18 (100%)
No or mild hydronephrosis, *n(%)*	17 (94%)
eGFR (mL/min/1.73 m^2^), median (range)	90(42–118)
Complications (Clavien Classification), *n(%)*	
Grade I	Urinary infection,4 (22.2%)
	Incision hernia, 1 (5.5%)
	Kidney stone formation, 4(22.2%)
	Metabolic acidosis, 6 (33.3%)
Grade II	Incomplete ileus, 4 (22.2%)
Grade IIIb	Incision infection,1 (5.5%)

BMI, body mass index; eGFR, estimated glomerular filtration rate

**Fig. 2:**
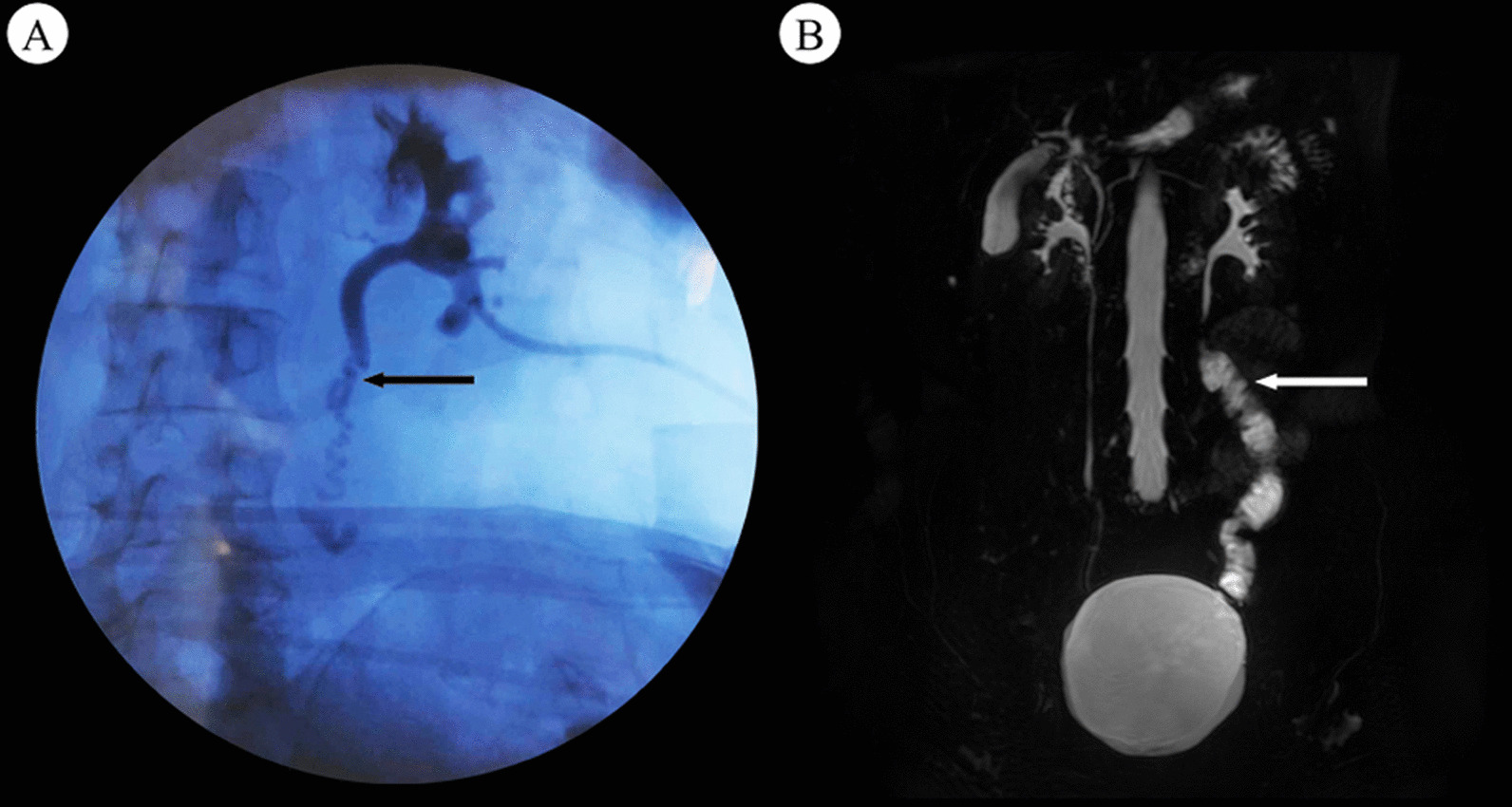
**A** The preoperative ureterography showed the location of the ureteral injury (black arrow). **B** Postoperative cine magnetic resonance urography (cine MRU) showed no severe hydronephrosis and the ileal ureter (white arrow)

A D-J stent was inserted intraoperatively in all surgeries and was removed in 2 months. All 18 patients were followed for a median of 16 (13–28) months. The median postoperative eGFR was 90 (42–118) ml/min/1.73 m^2^, which was stable. Compared with the preoperative ureterography findings (Fig. 2A), no or only mild hydronephrosis and excellent peristalsis of the ileal ureter were confirmed in 17 patients on postoperative cine MRU (Fig. 2B). One patient had moderate hydronephrosis based on ultrasound and no peristalsis of the distal ileum ureter was observed on cine MRU. The eGFR was stable and the patient remained under close observation without special treatment.

There were no intraoperative complications. Postoperatively, 1 major complication (Grade IIIb) occurred according to the Clavien–Dindo classification system [23]. The obese patient (BMI = 32 kg/m^2^) who had an incision infection responded well to thorough debridement under general anesthesia and regular bedside dressing changes. This patient had a long postoperative hospital stay (27 days). Four patients developed postoperative incomplete ileus (Grade II), leading to a delay in regular diet. All of these patients had an amelioration of symptoms with conservative therapy. Four patients had recurrent urinary infections that responded well to oral antibiotics (Grade I), 1 patient had a mild symptomatic incision hernia that remained stable without surgical intervention (Grade I), and the other patients were symptom-free. Metabolic acidosis was noted in 6 patients who required oral sodium bicarbonate (Grade I). Four patients developed kidney stones and agreed to conservative therapy (Grade I).

## Discussion

Since its introduction in 1959, ileal ureter replacement has been verified to be a feasible and safe surgical treatment option with good and durable outcomes in patients with long and complex ureteral obstruction [7]. The traditionally large incision in open procedures is always associated with a scar, more pain, and slower recovery. With increasing experience and expertise, urologists have begun performing these complex procedures using a laparoscope. A recent study demonstrated significant benefits in terms of narcotic analgesic use and convalescence, as well as a trend in shorter hospital stays for the laparoscopic group with satisfactory and efficacious outcomes [9].

Robotic platforms can be used to further facilitate the laparoscopic procedures, which allow for three-dimensional visualization and provide increased degrees of freedom that facilitate complex sutures. Moreover, ergonomics with robotic surgery is significantly less challenging both physically and cognitively [24, 25]. All of these factors make robotic platforms a good option for intracorporeal pyeloileal and ileovesical anastomoses, which are the most challenging aspects of ileal ureter replacement. The first robotic ileal ureter replacement procedure was performed by Wagner [10]. Isolation of the ileal segment, and pyeloileal and ileovesical anastomoses were performed intracorporeally, while re-establishing bowel continuity was performed extracorporeally. Repositioning the robot four times and passing the wire down the ileal segment intracorporeally might account for the 9-h operating time, according to the authors. There were no postoperative complications and the long-term outcome was encouraging.

With the development of endoscopic gastrointestinal staplers, the technical steps of ileal segment isolation and re-establishing bowel continuity can be completely performed intracorporeally. Thus, complete laparoscopic and robotic intracorporeal ileal ureter replacements were introduced by Sim and Brandao in 2014, respectively [11, 26]. Several studies have described their own technique considerations with low complication rates and encouraging functional outcomes [12–15]; however, repositioning, undocking, and redocking the robot cannot be omitted given the need to work in different abdominal compartments. Recently, Ubrig et al. used a 4-arm transperitoneal technique in 7 patients and undocking or redocking was not necessary [16]. However, a totally intracorporeal procedure is still a challenge for beginners, especially when re-establishment of bowel continuity is performed. Improper operations may lead to enteric anastomotic leakage and abdominal infections, which have been reported [9].

Based on our abundant experience with ileum ureteral replacement and > 100 robotic ureteral reconstructive surgical procedures, we designed a surgical technique involving robot-assisted laparoscopic ileal ureter replacement with extracorporeal ileal segment preparation. We have reported our experience of ileal ureteral replacement for the management of ureteral avulsion during ureteroscopy lithotripsy [27]. Manipulation of the ileal segment was performed extracorporeally, which has its own advantages. First, it can ensure accurate measurement and refashioning of the intestine to the needed length, which can be technically challenging and time-consuming intracorporeally [8]. It is unanimously agreed that the use of an excessive ileal segment size will increase the risk of postoperative metabolic and intestinal complications, hence an accurate measurement is critical. In addition, we made a 3-cm incision in both margins of the harvested mesenteric bowel segment to reduce radian which is our innovative modification (Fig. 1H) to straighten and make full use of the ileal segment. In cases in which the length of the ileal segment was not sufficient to reach the dome of the bladder, ileal ureter replacement combined with a psoas hitch was performed [21]. Second, it can ensure secure and expeditious bowel anastomoses, which may reduce the possibility of enteric anastomotic leakage, with only a minor cosmetic disadvantage. Moreover, it can prevent fecal contamination of the peritoneal cavity in cases of inadequate bowel preparation. A thorough lavage of the ileal graft with diluted povidone iodine may reduce bacteriuria and bacteremia, which cannot be performed intracorporeally. Fourth, although undocking and redocking of the robot, as well as dissection and suturing of small incisions, may partially extend the operative time, extracorporeal ileum isolation, and re-establishing bowel continuity are easy and save time, thus decreasing the total operative time. In our patient cohort, the operative time was shorter compared to previous reports, as shown in Additional file 3: Table S1. Note that this is only a rough comparison, considering it is a modern and longer series. In addition, the entire procedure was economical for patients with shorter operative times and less high-value medical consumables.

Short-term grade III and IV complications (< 30 days postoperatively) were noted in 27 cases in 105 patients (25.7%) in a previous ileal ureter replacement study [28]. In our series, an incision infection due to fat liquefaction occurred in one obese patient (5.5%) and no other major complications occurred. We attributed this result to the robotic platform, which allows for better visualization, meticulous dissection, and better pyeloileal and ileovesical anastomosis, as well as the application of extracorporeal ileal segment preparation.

In this series, we routinely used an anti-refluxing nipple technique for the ileovesical anastomosis, as we previously reported [29]. It remains to be determined whether the anti-reflux technique is necessary. Waldnerd et al. suggested that the anti-reflux procedure is not necessary because reflux appears to have no detrimental effect on renal function in adults with ileal ureters [30]. Xu et al. showed that the proximal anti-refluxing technique appears to be a reliable procedure [31]. In our patient cohort, postoperative cine MRU showed excellent peristalsis of the ileal ureter in 17 patients. None of the patients required a secondary intervention. The serum creatinine and eGFR remained stable during the follow-up period.

Although we have presented a series of cases involving robot-assisted laparoscopic ileal ureter replacement with extracorporeal ileal segment preparation, we still consider the total intra-corporeal technique as our eventual goal. The intra-corporeal technique provides a precision surgical procedure, while minimizing the trauma. However, at present, there are still concerns about intra-corporeal procedures, including inaccurate measurement of intestinal length and an increased risk of abdominal contamination. Complete intra-corporeal ileal ureter replacement will be our next step after the resolution of the above shortcomings.

Two important limitations of our study were the small sample size and the short follow-up period. This study did not provide better functional outcomes compared to laparoscopic or open techniques; however, it did provide a description of the surgical technique and our initial experience.

## Conclusions

Robot-assisted laparoscopic ileal ureter replacement with extracorporeal ileal segment preparation is safe, feasible, and effective for the treatment of long ureteral strictures, especially in high-volume tertiary referral centers with extensive robotic surgery experience capable of managing severe peri-operative complications.

## Supplementary Information

**Additional file 1.** The surgical technique of robotic ileal ureter replacement with extracorporeal ileal segment preparation for long ureteral strictures (Part 1).

**Additional file 2.** The surgical technique of robotic ileal ureter replacement with extracorporeal ileal segment preparation for long ureteral strictures (Part 2).

**Additional file 3: Table S1. **Summary of the reported case series for unilateral robot-assisted ileal ureter replacement.

## Data Availability

The datasets generated and/or analyzed during the current study are not publicly available due to privacy or ethical restrictions but are available from the corresponding author on reasonable request.

## Abbreviations

eGFR: Estimated glomerular filtration rate
RECUTTER: Reconstruction of the urinary tract: technology, epidemiology and result
cine MRU: Cine magnetic resonance urography
