# Supplementary material for: Robot-assisted laparoscopic ileal ureter replacement with extracorporeal ileal segment preparation for long ureteral strictures: a case series
Source: BMC Surg. 2022 Dec 21;22:435. doi: 10.1186/s12893-022-01885-5 (PMC9773509; doi:10.1186/s12893-022-01885-5)
Supplement: Supplementary file 3 — Additional file 3: Table S1. Summary of the reported case series for unilateral robot-assisted ileal ureter replacement. [file 12893_2022_1885_MOESM3_ESM.docx]

Table S1. Summary of the reported case series for unilateral robot-assisted ileal ureter replacement

| Study | No. of patients | Construction of ileal ureter  Completely intracorporal/Extracorporeal | Operation time/min | Blood loss/mL | Complications | Postoperative hospitalization |
| --- | --- | --- | --- | --- | --- | --- |
| Wagner JR (10) | 1 | Completely Intracorporal | 540 | Negligible | None | 5 |
| Brandao LF (11) | 1 | Completely Intracorporal | 420 | 50 | None | 4 |
| Sim A (12) | 1 | Completely Intracorporal | 320 | 50 | Migrated stent | 8 |
| Chopra S (14) | 3 | Completely Intracorporal | 450 | 200 | None | 9（2-12） |
|  |  | Completely Intracorporal | 540 | 100 | Grade IVa:1(small bowel necrosis) |  |
|  |  | Completely Intracorporal | 420 | 50 | None |  |
| Koenig JF (15) | 1 | Completely Intracorporal | 600 | 10 | None | 4 |
| Ubrig B (16) | 7 | Completely Intracorporal | 328±66 | - | None or grade I: 4 (paresthesia around trocar site);  Grade II: 2 (transient paralytic ileus) | 13.9±2.8 |
| Yee CH (17) | 1 | Completely Intracorporal | 285 | 50 | None | 14 |
| Present study | 18 | Extracorporeal | 248(170-450) | 50 (10-200) | Grade I: 2 (urinary infection, incision hernia)  Grade II: 4 (incomplete ileus)  Grade IIIb: 1 (incision infection) | 7(5-27) |
